# Unveiling the multifaceted roles of extracellular vesicles in cancer: insights from molecular imaging and engineering strategies: EVs and cancer: advances in molecular imaging

**DOI:** 10.3724/abbs.2025123

**Published:** 2025-08-08

**Authors:** Yuqin Teng, Gang Huang, Hao Yang

**Affiliations:** 1 School of Health Science and Engineering University of Shanghai for Science and Technology Shanghai 200093 China; 2 Shanghai Key Laboratory of Molecular Imaging Shanghai University of Medicine and Health Sciences Shanghai 201318 China

**Keywords:** extracellular vesicles, molecular imaging tracer technology, tumor treatment, clinical application

## Abstract

Extracellular vesicles (EVs), a class of nanoscale, membrane-bound vesicles secreted by various cell types, have emerged as rapidly advancing fields of research in recent years. This heterogeneous vesicle is a versatile carrier system for a variety of biomolecules, including proteins, nucleic acids, and metabolites. EVs play pivotal roles in intercellular communication, immune regulation, and disease pathogenesis, with particular implications for cancer biology. On the one hand, EVs promote tumor progression and metastasis by facilitating communication between cancer cells and their microenvironment. On the other hand, EVs carry noncoding RNAs, such as miRNAs and other regulatory RNAs, which directly modulate immune cell function or exert antitumor effects by influencing cancer cell proliferation and apoptosis. In addition to their biological roles, EVs show great potential as drug delivery systems because of their ability to be effectively taken up by target cells and stably deliver therapeutic payloads. In the context of cancer therapy, natural EVs demonstrate inherent therapeutic potential, particularly in targeting highly metabolically active organs. Furthermore, engineered EVs, which serve as both therapeutic vehicles and molecular imaging probes, have demonstrated significant potential for cancer theranostics. This review focuses on elucidating the dynamic changes and biological functions of EVs *in vivo*, with the aim of exploring the translational potential of EV-based molecular imaging and tracing technologies in cancer treatment. This work seeks to provide critical insights that may enhance the precision and efficacy of tumor therapies, offering a foundation for future clinical applications.

## Introduction

EVs represent a heterogeneous population of membrane-bound nanostructures that are actively secreted into various biological fluids. These evolutionarily conserved nanovesicles are ubiquitously produced by virtually all cell types and are naturally present in physiological fluids, including blood, saliva, urine, and breast milk. On the basis of their biogenesis pathways and size distribution, EVs can be classified into two primary schemes: size-based categorization and biogenesis-dependent subtyping. On the basis of their dimensional parameters, EVs can be broadly divided into small EVs (< 200 nm) and large EVs (> 200 nm). The biogenesis classification yields distinct subtypes, including exosomes (derived from multivesicular bodies), ectosomes (plasma membrane-derived), migrasomes (migration-dependent release), and apoptotic bodies (cell death-associated), among others [1]. In particular, small EVs, the smallest subtype characterized by their endosomal origin, serve as natural nanocarriers of diverse biomolecular cargo, including proteins, nucleic acids ( *e*. *g*., mRNAs, microRNAs, lncRNAs, circRNAs), and metabolites. The intricate combination of these biomolecular components confers dual functionality to EVs, serving as both crucial biological messengers in cellular communication networks and efficient natural carriers for targeted therapeutic applications.

**Figure 1:**
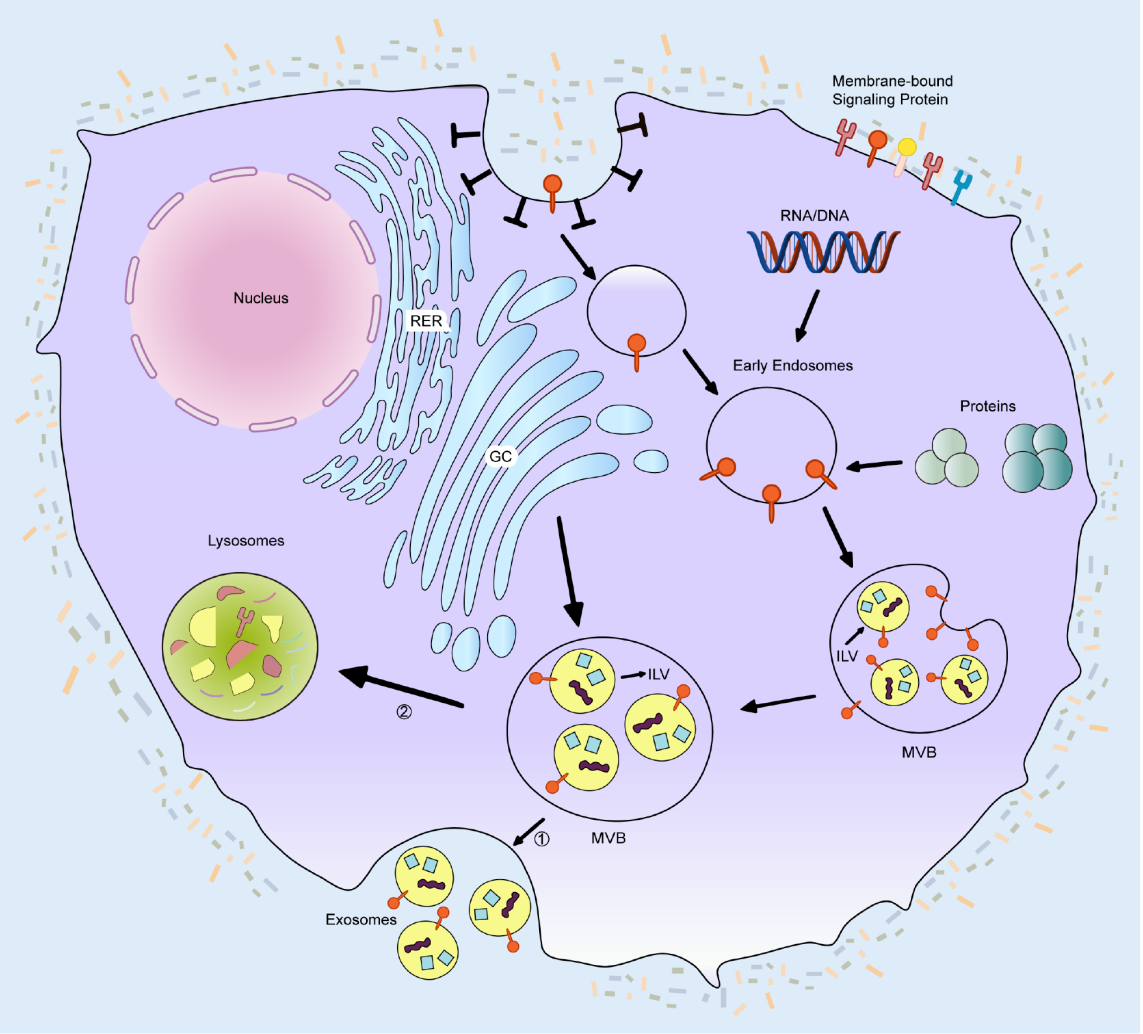
Figure 1 Biogenesis and secretion pathways of EVs EV biogenesis is initiated when membrane-anchored signaling proteins engage extracellular ligands at the plasma membrane. This ligand-receptor interaction induces clathrin-dependent endocytosis, resulting in membrane invagination and subsequent formation of an early endosome containing the ligand complex. Following scission from the membrane, the early endosome undergoes a maturation process characterized by progressive membrane invagination that selectively incorporates cytoplasmic constituents, including nucleic acids (DNA, RNA), proteins, and other macromolecules, into developing intraluminal vesicles (ILVs). At this point, late endosomes are called multivesicular bodies (MVBs). Mature MVBs migrate through the intracellular transport system to the plasma membrane, where they fuse and eventually release ILVs into the extracellular matrix. These secreted vesicles are known as EVs. RER, rough endoplasmic reticulum; GC, Golgi complex.

The biogenesis of EVs is initiated by invagination of the plasma membrane, which results in the formation of specialized microdomains that selectively incorporate extracellular components and membrane-associated molecules, thereby generating early endosomes. These early endosomes subsequently undergo a maturation process involving complex biochemical modifications and structural remodeling, ultimately transforming into multivesicular bodies (MVBs). During MVB formation, the limiting membrane of late endosomes buds inwardly, giving rise to intraluminal vesicles (ILVs) through a tightly regulated process. This crucial step is governed primarily by the endosomal sorting complex required for transport (ESCRT) machinery. The ESCRT system orchestrates the precise sorting and packaging of proteins, nucleic acids, and other biomolecules into ILVs, which constitute the fundamental cargo of future EVs. Notably, MVBs can undergo distinct trafficking fates: they may either fuse with the plasma membrane to release ILVs as EVs into the extracellular space or, alternatively, merge with lysosomes, leading to the degradation of their intraluminal contents through lysosomal enzymatic activity. This dual fate underscores the dynamic regulation of EV biogenesis and secretion ( Figure 1).

This review focuses on the analysis of the dynamic changes in EVs *in vivo* and explores the application value of EV molecular imaging tracer technology in tumor treatment in detail, aiming to lay a foundation for providing more accurate information on the effects of tumor treatment and important information.

## Role of Extracellular Vesicles in Tumor Occurrence and Development

EVs, which serve as crucial mediators of intercellular communication, transport diverse bioactive molecules, including noncoding RNAs, DNA, and proteins. These vesicles regulate recipient cell functions through autocrine and paracrine mechanisms, playing pivotal roles in tumorigenesis and progression. Emerging evidence indicates that EVs not only participate in tumor proliferation, metastasis, and drug resistance but also demonstrate significant potential in tissue repair and regeneration. With respect to tumor proliferation, Raimondo *et al*. [2] demonstrated that chronic myeloid leukemia-derived EVs promote tumor proliferation through autocrine mechanisms. Furthermore, as essential mediators of paracrine signaling, EVs have promising applications in tissue repair [3]. Chen *et al*. [4] employed human urine-derived stem cell (USC)-derived EVs to increase angiogenesis through the delivery of highly glycosylated polypeptide protein (DMBT1), suggesting novel therapeutic strategies for diabetic wound treatment. In the context of tumor drug resistance, EVs mediate resistance through multiple mechanisms, including facilitating cytotoxic drug efflux or transferring drug-resistant phenotypes via EV-mediated intercellular communication. Our previous study demonstrated that EVs secreted by cisplatin-resistant non-small cell lung cancer (NSCLC) cells under hypoxic conditions inhibit the PKM2-mediated apoptosis pathway, identifying both EVs and PKM2 as critical drivers of chemoresistance in NSCLC [5]. In terms of tumor metastasis, tumor-derived EVs facilitate cancer progression through multiple pathways, including epithelial-mesenchymal transition induction, proliferation promotion, apoptosis inhibition [6], immunosuppression, and enhancement of hematogenous metastasis and angiogenesis [7]. These multifaceted mechanisms establish EVs as indispensable regulators within the tumor microenvironment, offering novel perspectives for cancer diagnosis and therapeutic development.

### Tumor invasion and metastasis

The epithelial-mesenchymal transition (EMT) represents a fundamental cellular transformation process wherein epithelial cells acquire mesenchymal characteristics, serving as a critical mechanism in cancer metastasis. This complex biological process is regulated by multiple molecular factors, including E-cadherin suppression, transforming growth factor β (TGF-β) signaling, Wnt/β-catenin pathway activation, microRNA (miRNA) regulation, and specific transcription factors [ 8– 13] . During EMT activation, tumor cells undergo profound phenotypic changes and lose epithelial markers while gaining mesenchymal properties, thereby increasing their invasive capacity and facilitating the establishment of a metastasis-promoting microenvironment. Tumor-derived EVs have emerged as crucial regulators that modulate the tumor microenvironment and facilitate EMT progression. Recent investigations have demonstrated that colorectal cancer-derived EVs can orchestrate myeloid-derived suppressor cell recruitment through cancer-associated fibroblast (CAF) activation, subsequently impairing natural killer (NK) cell function and establishing an immunosuppressive tumor microenvironment [14]. Research from You *et al*. [15] revealed that CAF-derived EVs can transport zinc finger transcription factors (SNAIs) to lung cancer cells while concurrently releasing TGF-β and other cytokines, thereby inducing EMT through intracellular signaling pathway activation and promoting pulmonary metastasis. Furthermore, tumor EVs facilitate M2-type polarization of tumor-associated macrophages (TAMs) through EV cargoes and protein transfer. These M2 macrophages exhibit immunosuppressive properties through TGF-β and IL-10 secretion, further promoting tumor cell EMT. Recent advances have highlighted the intricate relationship between metabolic reprogramming and EMT regulation. Emerging evidence indicates that metabolic gene recombination influences EMT progression, with phosphorylated uridine diphosphate glucose interacting with human antigen R (HuR) to increase SNAL1 mRNA stability. This molecular interaction elevates SNAL1 expression levels, thereby promoting EMT activation and facilitating distant tumor metastasis [16]. As a pivotal biological process in tumor progression, EMT involves multiple interconnected signaling pathways and molecular mechanisms, offering valuable insights for developing novel therapeutic strategies and improving clinical outcomes.

### Proliferative and antiapoptotic effects

Tumor-derived EVs significantly contribute to cancer metastasis by modulating cellular proliferation and apoptotic pathways [17]. Huang *et al*. [18] demonstrated that non-small cell lung cancer (NSCLC)-derived EVs enhance proliferation in both normal lung fibroblasts and NSCLC cells through α-smooth muscle actin (α-SMA) delivery while simultaneously suppressing apoptosis. In addition to protein transfer, EV-encapsulated miRNAs play crucial regulatory roles in these processes. Kruppel-like factor 10 (KLF10), a zinc-finger transcription factor, serves as a key regulator of cellular growth, apoptosis, and differentiation. Chen *et al*. [19] reported elevated expression of miR-340-5p in hypoxic oral squamous cell carcinoma (OSCC)-derived EVs, which are transferred to normoxic OSCC cells and directly target the tumor suppressor KLF10, thereby promoting apoptosis. These findings collectively demonstrate that tumor EVs facilitate metastatic progression through the targeted delivery of specific proteins and miRNAs that coordinately regulate proliferation and apoptosis.

### Tumor immune regulation

EVs serve as pivotal mediators in tumor immunology by facilitating intercellular communication between tumor cells and immune cells, thereby modulating tumor initiation and progression. Notably, mesenchymal stem cell-derived EVs (MSC-EVs) exhibit significant immunomodulatory effects through T-cell regulation, macrophage polarization, and angiogenesis control [ 20– 22] . Experimental evidence indicates that under pathological conditions, MSCs-EVs attenuate autoimmune and inflammatory disease progression by suppressing Th1-type immune responses. Specifically, Li *et al*. [23] reported that MSC-EVs reduce Th1/Th17 cell populations while expanding regulatory T cells (Tregs), concurrently ameliorating lupus nephritis through B-cell response inhibition. Guo et al. [24] further established that MSCs-EVs suppress contact hypersensitivity via Th1 cell activity inhibition. Additional studies [25] revealed that MSC-EVs induce Th1-to-Th2 cell polarization, consequently decreasing the levels of proinflammatory cytokines ( *e*. *g*., IL-1β and TNF-α) while increasing the levels of anti-inflammatory mediators. Spleen-derived EVs demonstrate enhanced immunoregulatory capacity in hepatocellular carcinoma [26] and gastric cancer [27] models, whereas lymph node-derived EVs contribute significantly to tumor immunotherapy [28] and the delivery of immunostimulants [29]. Immune cell-derived EVs orchestrate multifaceted antitumor immunity through direct antigen presentation to T/B cells [30] or indirect presentation via dendritic cell (DC) transfer [31]. Crucially, DC-derived EVs outperform tumor-derived EVs in immunostimulatory potency [32], as DCs package diverse immunogenic components (MHC-peptide complexes, costimulatory molecules, cytokines) within their EVs, enabling robust T-cell/NK cell activation and potent antitumor responses. Furthermore, immune cell-derived EVs activate NK cells and macrophages, amplifying immunostimulation. Collectively, EVs present multidimensional therapeutic potential in cancer immunotherapy, functioning both as efficient antigen-presenting vehicles and immune microenvironment modulators ( *e*. *g*., via immune cell activation and immunosuppressive cell inhibition). These properties establish EVs as promising platforms for developing next-generation immunotherapies.

## Role of Extracellular Vesicles in Tumor Diagnosis and Treatment

### Tumor monitoring markers

EVs are emerging as key players in tumorigenesis and progression, thereby establishing a strong foundation for their potential applications in diagnostic and therapeutic strategies. Given their rich cargo of diverse biomolecules, EVs hold significant promise as diagnostic biomarkers for tumor detection. These biomolecules can be broadly classified into several categories: (1) EV nucleic acids, such as miRNAs [33], mRNAs, IncRNAs, and DNAs, among which miRNAs have great advantages in tumor diagnosis. Fang *et al*. [34] reported that EVs carrying miR-103 in hepatocellular carcinoma cells directly inhibited VE-cadherin (VE-Cad), p120-catenin (p120) and zonula occludens 1 expressions to attenuate the integrity of endothelial junctions and promote tumor cell metastasis, suggesting that EVs carrying miR-103 could be used as potential therapeutic targets and markers for predicting the metastasis of liver cancer. (2) EV proteins include the cell surface proteoglycan glypican-1 (GPC1), which is enriched in EVs, and Melo *et al*. [35] reported that GPC1+crExos can be detected in the serum of pancreatic cancer patients with absolute specificity and sensitivity and therefore can be used as a means of distinguishing among healthy individuals, patients with benign pancreatic diseases and early and advanced pancreatic cancer patients. Ogata-Kawata *et al*. [33] designed experiments to identify several EV miRNAs that appeared to serve as markers reflecting pathological changes in colorectal cancer patients by comparing the sera of colorectal cancer patients with those of healthy controls. (3) EV lipids and small-molecule metabolites. Skotland *et al*. [36] demonstrated the potential of EV lipid species in urine as biomarkers for prostate cancer. (4) In EVs, cyclic RNA has a unique covalently closed loop structure that makes them highly resistant to nucleic acid exonucleases and therefore stable in EVs. Meng *et al*. [37] demonstrated that tumor-derived circSCP2 can sponge miR-92a-1-5p and interact with PTBP1, thereby stabilizing IGF2BP1 expression and enhancing tumor cell proliferation, invasion and metastasis.

### Tumor treatment tools

EVs play a complex role in the tumor growth process, and their role is dual: on the one hand, they can promote tumor growth and metastasis, and on the other hand, they may inhibit tumor progression. This dual role makes EVs important objects in tumor therapy research. As a tumor therapeutic tool, EVs function mainly through various mechanisms, such as immune activation, tumor microenvironment regulation, induction of apoptosis, inhibition of angiogenesis, and inhibition of metastatic ability.

In terms of immune activation, some types of EVs are able to load tumor antigens and effectively enhance the activity of immune cells ( *e*. *g*., dendritic cells and T cells), thus increasing the recognition and attack ability of the immune system against tumor cells. Lyu *et al*. [38] reported that certain T-cell-derived EVs activate the immune activity of immune cells, and Wang *et al*. [39] reported that EVs derived from phosphoantigen-expanded Vδ2-T cells were able to induce apoptosis in EBV-associated gastric cancer cells and successfully inhibited the growth of EBV-associated gastric cancer in a Rag2 ^–/–^γc ^–/–^ mouse model; in terms of tumor microenvironment regulation, EVs can inhibit tumor growth and metastasis by regulating the TME. For example, EVs can inhibit the activity of CAFs and immunosuppressive cells, thus weakening the immunosuppressive effect in the tumor microenvironment and reducing tumor growth and metastasis; in terms of apoptosis induction, specific types of EVs can carry relevant factors that induce apoptosis in tumor cells. For example, certain miRNAs ( *e*. *g*., miR-34a) in EVs can inhibit tumor cell proliferation and induce apoptosis [40]; in terms of angiogenesis inhibition, EVs are able to release factors that inhibit angiogenesis, thereby suppressing tumor growth. In addition, EVs can also transport RNA or protein components of oncogenes, which can stimulate oncogenic effects after being taken up by tumor cells and ultimately inhibit tumor cell proliferation and growth [14]. In terms of metastatic ability inhibition, EVs can intervene in the migratory and invasive ability of tumor cells, thus effectively reducing their metastatic potential. Wiklander *et al*. [41] were able to reduce the tumor burden and prolong the survival of mice with subcutaneous melanoma via systemic injection of EVs displaying PD-L1 antibodies loaded with the chemotherapeutic drug doxorubicin.

## Isolation and Purification of Extracellular Vesicles

The pivotal role of EVs in tumorigenesis, development, and diagnosis has been extensively validated. To further elucidate their biological functions and advance clinical applications, the development of highly efficient and high-purity EV isolation techniques is of paramount importance. Currently, ultracentrifugation is considered the gold standard for EV isolation because of its consistent separation efficacy [42]. However, traditional methods often face technical limitations, such as cumbersome procedures and prolonged processing times. To address these issues, researchers have developed the polyethylene glycol (PEG) precipitation method, which significantly reduces the separation time. Nevertheless, subsequent studies revealed limitations, including low recovery rates, insufficient purity, and challenges in effectively removing contaminating impurities [43]. In the pursuit of improved purity, microfluidic-based separation technologies have emerged. Compared with conventional methods, microfluidic systems leverage precise and controllable microscale hydrodynamic properties to achieve more refined control over EVs. Additionally, microfluidic systems offer high automation capabilities, enabling the integration of complex multistep separation processes onto a single chip. This not only minimizes manual operational errors but also substantially reduces the separation time. However, microfluidic technology faces challenges, including high equipment costs, complex chip fabrication processes, and stringent technical expertise requirements, which limit its widespread adoption. Despite these limitations, ongoing advancements in biotechnology are expected to overcome these bottlenecks. Microfluidic separation technology is anticipated to complement ultracentrifugation, PEG precipitation, and other isolation methods, collectively advancing EV research toward increased precision and efficiency ( Table 1).

**Table:** **Table 1** Recovery rates, purities, and application scenarios of different separation techniques

Separation technology	Clinical recovery rate	Laboratory purity	Application scenario	Ref.
Ultracentrifugation	Low recovery	High purity achievable via optimized conditions	Gold standard, ideal for processing large sample volumes	[ 44– 46]
Size exclusion chromatography	Relatively low	High purity	Applicable to a broad range of research contexts	[ 47– 49]
Polyethylene glycol precipitation	High recovery	Low purity	Applicable to a broad range of research contexts	[ 50, 51]
Ultrafiltration	High recovery	Low purity	Well-suited for large-scale sample processing	[ 52– 54]
Microfluidics	High recovery, 87%	High purity, 97%	Better suited for clinical diagnostic use with small sample sizes	[ 55– 57]

### Ultracentrifugation (UC)

In 1920, Swedish chemists Svedberg and Rinde constructed the world’s first ultracentrifuge and successfully isolated hemoglobin, marking a significant advancement in separation technology [44]. Currently, ultracentrifugation has become the most widely used laboratory method, which relies on the principle that particles sediment at different rates under varying centrifugal forces. Initially, the centrifuge operates at low speeds, allowing larger particles and dead cells to settle at the bottom of the tube. The speed subsequently increases to precipitate smaller particles, resulting in higher-purity EVs. This method offers notable advantages, including the ability to separate high-density and low-density EVs and suitability for large-scale sample processing. However, it also has limitations, such as the need for multiple centrifugation steps, labor-intensive procedures, poor recovery of EVs from high-viscosity biological samples, and the significant impact of centrifugation speed on yield and purity [45]. To address these limitations, density gradient centrifugation was developed. This technique utilizes separation media with varying densities, such as sucrose and iodixanol, to achieve separation on the basis of differences in particle sedimentation rates. D′Acunzo *et al*. [46] compared sucrose and iodixanol density gradients and reported that while the total EV yield was similar between the two, iodixanol gradients provided more precise differentiation of EV subtypes. In laboratory settings, both density gradient centrifugation and differential centrifugation are commonly employed, each with distinct advantages and limitations. Density gradient centrifugation offers higher resolution and cleaner separations but is more complex and time-consuming. In contrast, differential centrifugation is simpler and suitable for rapid preliminary separations, although its separation efficiency is inferior to that of density gradient centrifugation. Therefore, the choice of centrifugation method should be guided by experimental objectives and sample characteristics.

### Size exclusion chromatography (SEC)

Size exclusion chromatography is a widely employed technique for separating molecules on the basis of their size differences. It has demonstrated excellent performance in terms of purity and recovery. It is widely used in the extraction and purification of EVs [47]. The separation principle relies on the differential behavior of molecules as they flow through an SEC column packed with porous material: small molecules ( *e*. *g*., proteins, lipids) are more likely to enter the pores and become adsorbed, whereas larger molecules or particles ( *e*. *g*., EVs), which cannot enter the pores, are eluted by the buffer and collected in the sample tube. This size-based separation mechanism gives SECs a unique advantage in isolating EVs. In their study, Alinda *et al*. [48] successfully isolated EVs from the adipose-derived stem cell stromal vascular fraction (AD-SVF) via SEC and characterized the isolated products via nanoparticle tracking analysis (NTA). The results demonstrated that SEC efficiently isolates EVs of specific sizes and concentrations. Furthermore, Bai *et al*. [49] reported that tandem SEC outperformed conventional single SEC in terms of product purity. Notably, when combined with ultracentrifugation, SEC showed significant advantages in both the total number of proteins identified and the overlap with the top 100 EV markers. However, SEC has limitations when processing complex samples. Since its separation principle is primarily based on size differences, it struggles to exclude impurities with similar particle sizes, resulting in contamination by heteroproteins and limiting purity. Additionally, the adsorption and elution properties of SEC columns become imbalanced when they are overloaded with samples, leading to reduced separation efficiency. Moreover, SEC columns have strict limits on the injection volume, and exceeding these limits can cause column overloading, severely compromising separation performance. Therefore, while SEC holds significant value in EV separation, further optimization is needed to increase its applicability for complex samples.

### Polyethylene glycol (PEG) precipitation

Owing to its unique chemical properties, polyethylene glycol has a wide range of roles in various fields, particularly in drug release. For example, Zheng *et al*. [51] proposed a novel bone-targeted drug delivery system. The system uses PEGylated carboxylic acid-modified polyamide amine dendritic macromolecules, which significantly enhance the targeting and therapeutic efficacy of drugs in bone. Ludwig *et al*. [50] reported that the PEG method resulted in higher yields and purities of EVs. The mechanism of action of the PEG method for the extraction of EVs is as follows: PEG, as a water-soluble nonionic compound, is strongly hydrophilic and can bind to the hydrophobic lipid bilayer, thereby changing the solubility of EVs and causing them to precipitate. The specific protocol involves the collection of cell culture medium containing EVs, followed by pretreatment of the sample and filtration to remove particulate matter. The sample is subsequently mixed with a specific concentration of PEG and stirred at the appropriate temperature to allow the PEG to combine with the EVs to form a complex. The complexes of PEG and EVs were then separated via centrifugation. Finally, the complexes are then washed with buffer to remove PEG residues, and anticoagulants can be added during the washing process to prevent the EVs from agglomerating. The advantages of this method include its simplicity, lack of specialized equipment requirements, and low cost. However, a notable disadvantage is the potential coprecipitation of non-EV hydrophobic substances, which can compromise EV purity.

### Ultrafiltration (UF)

Ultrafiltration is a separation technology based on the principle of molecular size exclusion, the core of which is the use of a filter membrane with a specific pore size to achieve accurate screening of EVs. In experimental operations, researchers typically employ a hierarchical filtration strategy: first, a micron-sized pore size prefilter is used to remove large particles such as cell debris to obtain a primary filtrate rich in EVs; subsequently, nanosized ultrafiltration membranes are used for fine separation, and vesicles smaller than the target size are excluded from the waste solution. Recent studies have demonstrated that tangential flow filtration (TFF), a membrane-based ultrafiltration technique, significantly enhances the isolation and purification of EV-mimetic nanovesicles (NVs) when combined with freeze-thaw processing [52]. Compared with naturally derived EVs, this integrated approach yields substantially greater quantities of NVs while maintaining their structural and functional integrity. UF is a relatively simple and low-cost method for separating EVs that does not require expensive equipment and is relatively easy to operate. Second, compared with ultracentrifugation, the UF method is more suitable for the processing of large-scale samples and requires less time. The disadvantage is that the EVs extracted from UF may be significantly contaminated with non-EV-free proteins, such as α1-antitrypsin [53] and albumin [54]. On the other hand, the interaction of EVs with the filter membrane is highly likely to promote the formation of polymers on the membrane between the EVs and the proteins in the solution, clogging the pores and reducing the efficiency of UFs, thereby reducing the purity and yield of the separated EVs.

### Microfluidics

Microfluidics, as a cutting-edge scientific tool that focuses on the precise manipulation of microscale fluids, deeply integrates multidisciplinary knowledge of chemistry, fluid physics, microelectronics, new materials, biology, and biomedical engineering and is representative of the emerging cross-disciplinary field. Qian *et al*. [56] constructed a microfluidic magnetic detection system based on DNA tetrahedral structured probes (TSPs) (μFMS) that combines microfluidic technology and magnetic detection to achieve rapid detection of tumor-derived EVs (TDEs). The quantitative detection of EV concentration is achieved by accurately determining the relative impedance change value generated by the microelectrode system after capturing TDEs. Meng *et al*. [55] conducted a comprehensive evaluation of the device using whole blood samples. The results demonstrated that the device could efficiently isolate fluorescently labeled small extracellular vesicles with purities exceeding 97% while maintaining a high recovery rate of 87%. Furthermore, Zhao *et al*. [57] devised a fully integrated microfluidic chip capable of automating the isolation of EVs directly from whole blood samples. This study also established a comprehensive platform integrating on-chip EV isolation and *in situ* detection, effectively differentiating cancer patients from healthy individuals. Currently, microfluidic technology still faces many technical bottlenecks in the field of EV detection. On the one hand, there is significant biological heterogeneity in EV samples, and the complex biological background interference and significant differences in the expression profiles of miRNAs between different subpopulations of EVs seriously affect the specificity and accuracy of detection; on the other hand, the magnetic microfluidic chip has a relatively single function, which restricts the application scenarios; in addition, the sensitivity of the detection signal amplification system still needs to be improved, and how to realize the significant enhancement of the detection signal is also a major challenge. These challenges indicate that the application of microfluidic technology in EV detection still needs to be explored and optimized at a deeper level in terms of chip design, signal amplification and bioinformation processing.

## Molecular Imaging Tracing Techniques for Extracellular Vesicles

**Figure 2:**
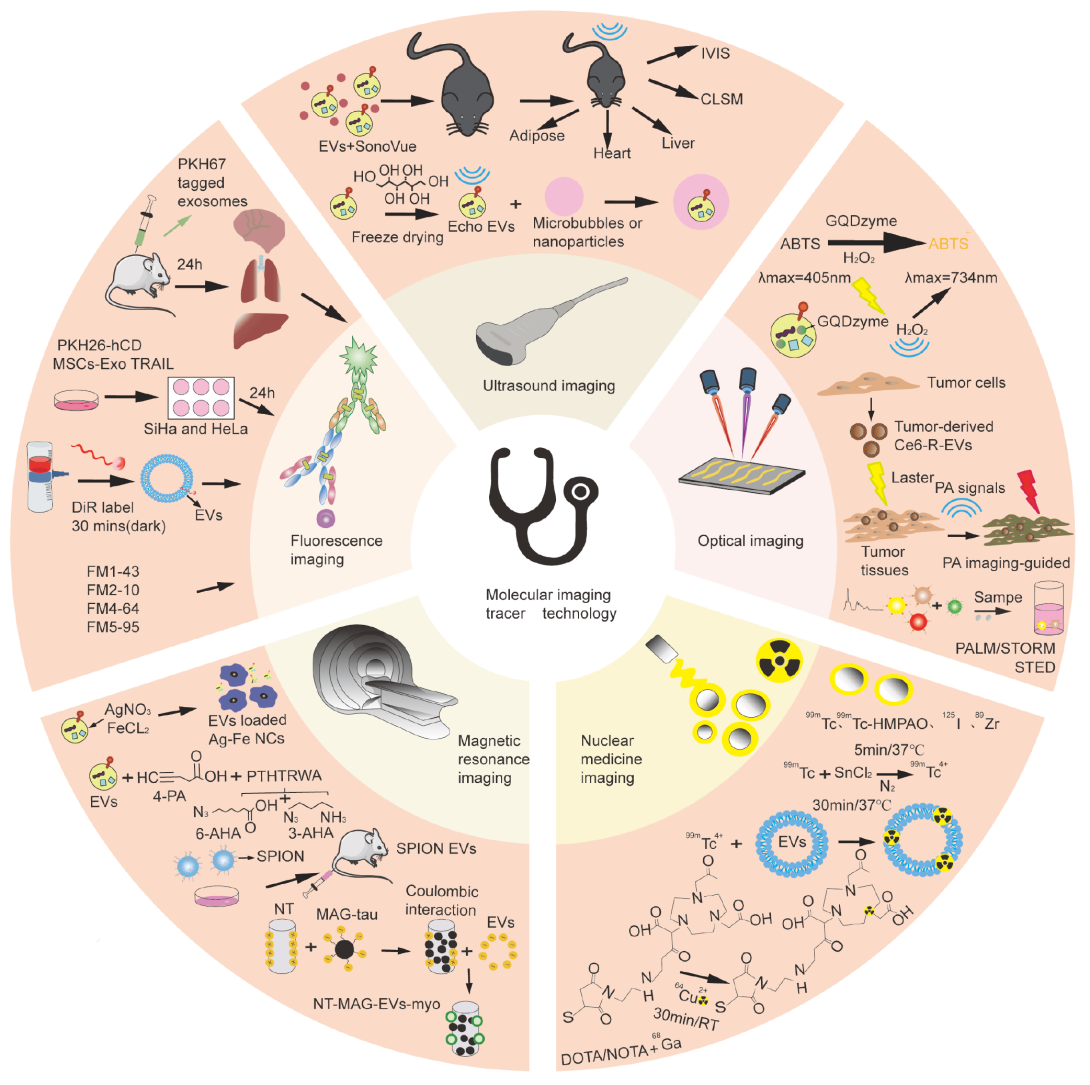
Figure 2 Applications of EVs in molecular imaging techniques, including fluorescence imaging, ultrasound imaging, photoacoustic imaging (PAI), magnetic resonance imaging (MRI), and nuclear medicine imaging Fluorescence imaging was used to track the biodistribution of PKH67-labeled EVs in mouse organs (heart, lung, spleen, liver, and nervous system) at 24 hours postinjection. Cellular uptake was evaluated using PKH26-labeled hCD-MSC-EVs or hCDMSC-EVsTRAIL cocultured with cervical cancer cells (SiHa and HeLa) for 24 hours. Short-term EV tracking employed DiR labels (30 minutes of incubation in the dark), while membrane labeling used FM-series dyes (FM1-43, FM2-10, FM4-64, and FM5-95). Ultrasound imaging combines SonoVue with EVs or utilizes freeze-dried, mannitol-stabilized cow milk-derived EVs (“echo-EVs”) for enhanced acoustic contrast. Photoacoustic imaging leverages GQDzyme/ABTS as tracers for tumor visualization or dihydroporphyrin E6-loaded EVs for enhanced signal generation; when Raman imaging is performed for the detection of specific EV subtypes, three distinct SERS probes are mixed with the sample. Upon the binding of target EVs, an aptamer-immune complex forms between the EVs, the substrate, and the corresponding SERS probe, enabling selective identification and signal amplification. superresolution microscopes include PALM/STORM and STED. Magnetic resonance imaging has partially employed Ag/Fe₃O₄ nanoclusters as self-assembled dual-modal probes on EVs. Alternative approaches include PTHTRWA-based probes with C-/N-terminal EV functionalization and ferromagnetic nanotubes (NT-MAGs) for magnetic-guided EV delivery. Nuclear medicine imaging has demonstrated in vivo EV tracking through direct radiolabeling or radionuclide conjugation via bifunctional chelating agents (BFCAs).

To investigate the physiological functions, metabolic processes, and distribution of EVs *in vivo*, effective tracer technologies are essential. As a crucial tool for studying EVs, tracer technology enables real-time and dynamic observation of EV activity both intracellularly and extracellularly. Common methods include fluorescence imaging, magnetic resonance imaging (MRI), nuclear medicine imaging, optical imaging, and ultrasound imaging. With the growing demand for precise imaging in medical research, multimodal imaging is achieved by integrating fluorescence imaging, computed tomography (CT), magnetic resonance imaging (MRI), photoacoustic imaging (PAI), and other techniques, thereby enhancing image contrast and providing richer imaging details. This section reviews the principles, advantages, and limitations of different molecular tracer technologies, as well as the current applications of EVs, on the basis of extensive related studies, to further advance EV research in basic medicine and clinical practice ( Figure 2).

### Fluorescence imaging

Fluorescence imaging holds a critical position in molecular and cellular biology as a widely adopted tracing technique. It is implemented primarily through two approaches. First, fluorescent proteins ( *e*. *g*., green fluorescent protein [GFP] and red fluorescent protein [RFP]) are fused with EV membrane markers (such as CD63, CD9, and CD81) to track EV movement precisely *in vivo*. Second, lipophilic fluorescent membrane dyes are incorporated into the EV lipid bilayer; when activated, these dyes convert optical signals into electrical signals via photomultiplier tubes, which are then visualized by imaging systems ( *e*. *g*., CCDs) for EV labeling and tracking. Commonly used dyes include PKH67, PKH26, Di series, and FM-64.

Fluorescent dyes can be functionally categorized into three groups. The first group includes PKH67 (green fluorescence) and PKH26 (red fluorescence), which are more widely used because of their better staining effect, as they can bind stably to the lipid bilayer membrane of EVs. PKH67-labeled EVs can be effectively used to track the behavior of EVs in the tumor microenvironment [58]. In addition, Ye *et al*. [59] cocultured hCD-MSC-EVs or hCDMSC-EVsTRAIL with cervical cancer cells (SiHa and HeLa) for 24 hours after they were labeled with PKH26 and observed that red fluorescence was concentrated in the nucleus via confocal fluorescence microscopy, which successfully confirmed that cervical cancer cells are sensitive to hCD-MSC-EV and hCD-MSC-EVTRAIL uptake. Rau *et al*. [60] cocultured PKH26 fluorescently labeled ADSC-EVs with the sciatic nerves of mice via confocal fluorescence microscopy and reported that the labeled EVs were able to be taken up by spinal cord neurons and proximal nerve segments, which indicated that the ADSC-EVs could reach damaged nerve regions. The second group comprises a series of lipophilic dyes, in which deep red fluorescence (DiR) penetrates cells and tissues during imaging and is therefore frequently employed for *in vivo* tracing. Santelices *et al*. [61] achieved a process from precollection of fine cultures to labeling of EVs with DiR dyes, which is essential for subsequent monitoring of EVs. The third group, the FM series [62], consists of four fluorescent dye products, FM1--43, FM2--10, FM4--64 and FM5--95. These dyes belong to the lipophilic styryl group, are nontoxic to cells, are mainly used for specific labeling of cell membranes in living cells, and are useful for tracking cytophagy and cytotoxicity. Fluorescence staining offers advantages such as operational simplicity and real-time, noninvasive analyte tracking. However, limitations include potential false-positive signals due to nonspecific liposome staining, the inability to accurately reflect the half-life of EVs [63], and restrictions to purified EVs from conditioned media or body fluids, excluding primary cell applications.

### Magnetic resonance imaging (MRI)

MRI, a radiation-free modality with superior soft-tissue resolution and multiplanar imaging capabilities, plays a crucial role in medical diagnostics. However, conventional MRI has limited sensitivity in detecting EVs because of their nanoscale dimensions and inherent low contrast. To enhance EV detection, researchers have developed magnetic contrast agent-based labeling strategies, with superparamagnetic iron oxide nanoparticles (SPIONs) being particularly promising owing to their exceptional magnetic properties. Recent studies have advanced MRI-based EV detection methodologies. Tayyaba *et al*. [64] developed *in situ* biosynthesized silver-iron oxide (Ag-Fe _3_O _4_) nanoclusters from HepG2 cells that function as both fluorescent probes and CT/MRI contrast agents, enabling multimodal cancer imaging through self-assembly of EVs. Kowalczyk *et al*. [65] engineered EVs by functionalizing their surfaces with PTHTRWA heptapeptide ligands to load SPIONs, demonstrating successful tumor targeting in mice following intravenous administration. Despite these advances, MRI-based EV detection faces technical challenges. First, limited sensitivity necessitates high marker concentrations for detectable signal changes. Busato *et al*. [66] proposed that efficient MRI detection can be achieved by using ultrasmall superparamagnetic iron oxide nanoparticles (USPIOs) to label EVs. Second, although MRI has excellent soft tissue contrast, its spatial resolution is still insufficient at the nanoscale, making it difficult to accurately present the morphological features and spatial localization information of EVs. To overcome these limitations, researchers have continued to explore new technological options, and Jung *et al*. [67] developed hypoxia-targeted EVs with *in vivo* tracking via magnetic particle imaging (MPI). Additionally, Villa *et al*. [68] proposed the use of ferromagnetic nanotubes (NT-MAGs) for magnetic field-guided EV delivery to specific muscle tissues, representing a novel targeting approach.

### Nuclear medicine imaging

The localization accuracy of EVs can be enhanced through radionuclide administration (injection or oral) combined with SPECT/PET-CT/MRI multimodal imaging. Current labeling methods primarily employ direct or indirect radionuclide incorporation. The direct approach uses radioisotopes ( *e*. *g*., ^99m^Tc, ^99m^Tc-HMPAO [69], ^125^I [70], and ^89^Zr [71]) to label EVs, enabling precise biodistribution tracking through radiometric detection. González *et al*. [72] pioneered diagnostic applications using ^99^mTc-labeled milk-derived EVs and demonstrated through SPECT imaging that intravenously, intraperitoneally, or intranasally administered EVs preferentially accumulated in hepatic and splenic tissues. Compared with intraluminal approaches, comparative studies by Faruqu *et al*. [73] revealed superior labeling efficiency and stability in membrane-labeled EVs. Notably, Almeida *et al*. [74] achieved breakthrough sensitivity in metastasis detection by labeling osteosarcoma-derived EVs with copper-64 ( ^64^Cu), identifying micrometastatic lesions as small as 2–3 mm via PET imaging. For indirect labeling, bifunctional chelators (DOTA/NOTA) form stable complexes with radionuclides such as ^68^Ga for EV conjugation. While these techniques show promise, critical challenges remain regarding the temporal differences between short EV half-lives and prolonged radionuclide activity—a disparity that may generate false-positive signals. Method-dependent variations in labeling efficiency and stability necessitate careful protocol optimization, and the inherent radiation risks mandate stringent dose control measures throughout experimental procedures.

### Optical imaging

Optical imaging technology is widely applied in biomedical research, particularly for the detection, characterization, and functional analysis of EVs. Techniques such as fluorescence imaging, PAI, Raman imaging, and superresolution microscopy offer powerful tools for EV studies.

PAI is an emerging modality that combines the principles of ultrasound and optical imaging through the photoacoustic effect. This technique operates by detecting acoustic waves generated when contrast agents in biological samples absorb pulsed laser energy. PAI uniquely merges the high spatial resolution and tissue penetration depth of ultrasound with the superior contrast of optical imaging [75]. Its application in oncology has been demonstrated in breast cancer studies, where intravenously injected EVs were successfully tracked via PAI, confirming its efficacy in monitoring tumor dynamics [76]. In further advancing therapeutic applications, Jang *et al*. [77] engineered EVs loaded with dihydroporphyrin E6, enabling simultaneous photoacoustic visualization and photodynamic therapy through targeted reactive oxygen species generation in tumor cells upon laser irradiation. Despite these advantages, PAI-based EV tracking faces several challenges that warrant further investigation. Key areas for improvement include standardization of imaging parameters, optimization of contrast agents, and enhancement of signal stability. Addressing these limitations will be crucial for maximizing the potential of PAI in EV research and clinical applications.

Moreover, surface-enhanced Raman spectroscopy (SERS) has also been applied for EV detection. This analytical technique integrates Raman spectroscopy with imaging technology, enabling the visualization of chemical spatial distributions within samples. By detecting variations in Raman spectral intensity or peak shifts across different sample regions, SERS can generate two- or three-dimensional chemical composition maps. Wang *et al*. [78] developed a specialized SERS probe combined with a machine learning algorithm, successfully distinguishing EVs derived from various cancer types. Similarly, Park *et al*. [79] fabricated laser-ablated silver nanoparticles (LA-AgNPs) as SERS substrates, identifying distinct spectral signatures to classify EVs by origin. Unlike conventional methods, SERS is nondestructive, preserving sample integrity while accommodating diverse EV analyses. However, its limitations include high equipment costs, a lack of standardization, challenges in large-scale validation, and insufficient detection throughput, which hinders comprehensive EV component analysis.

Recent advances in superresolution imaging have overcome the optical diffraction limit, permitting visualization of individual EVs and precise mapping of surface protein localization, which are critical for functional EV studies. This technology primarily employs two approaches. First, single-molecule localization microscopy ( *e*. *g*., PALM/STORM), in which photoswitchable fluorophores enable stochastic activation of sparse molecules, is used. After thousands of imaging cycles with nanometer-scale precision, the reconstructed coordinates yield a superresolved image. Second, stimulated emission depletion (STED) microscopy, which physically confines fluorescence emission via a donut-shaped depletion beam, effectively narrows the excitation spot for enhanced resolution. Chen *et al*. [80] demonstrated the utility of PALM/STORM in tracking cancer-derived EVs into recipient cells with high specificity, highlighting its potential for detecting EV membrane markers ( *e*. *g*., PD-L1 and HER2). Nevertheless, superresolution imaging faces constraints, including slow acquisition, high costs, and limited axial resolution [81]. Future efforts should address these shortcomings while refining novel methodologies to advance EV applications in biomedicine.

### Ultrasound imaging techniques

Ultrasound imaging has become an indispensable modality in modern medicine, integrating both diagnostic and therapeutic capabilities with widespread clinical applications. As a diagnostic tool, ultrasound uses high-frequency sound wave reflection to visualize the morphology and structure of internal organs and tissues with exceptional clarity. This technology demonstrates particular sensitivity in detecting subtle abnormalities in hepatic [82], gallbladder [83], uterine [ 84, 85] , and fetal [86] tissues while also enabling postoperative treatment evaluation [ 87, 88] . Through color Doppler technology, ultrasound further provides hemodynamic assessment, precisely identifying vascular pathologies such as obstructions and valvular regurgitation [89]. Recent advances have significantly enhanced ultrasound-based EV monitoring. Sun *et al*. [90] employed ultrasound-targeted microbubble destruction (UTMD) technology to create transient tissue channels that facilitate enhanced EV penetration across biological barriers. In an innovative approach, Osborn *et al*. [91] combined the acoustic properties of microbubbles with the low immunogenicity and nanoscale size of EVs, developing novel “echo-EVs” through freeze-drying bovine milk-derived EVs with mannitol. Current methodologies typically improve EV detectability by conjugating them with ultrasound contrast agents ( *e*. *g*., microbubbles or nanoparticles), enabling real-time noninvasive monitoring of their biodistribution, migration patterns, and tissue accumulation. This approach not only advances our understanding of EV biology but also creates new opportunities for therapeutic development and targeted drug delivery systems. Despite these advantages, technical limitations persist. Conventional microbubble contrast agents remain restricted to intravascular applications because of their relatively large size, highlighting the need for further optimization of ultrasound-based EV tracking methodologies.

## Molecular Imaging Targets for Labeling Extracellular Vesicles and Engineering Strategies

Recent studies have identified numerous proteins on EVs, including tetraspanins (CD9, CD63, and CD81), heat shock proteins (HSP70 and HSP90), cytoskeletal components (actin and microtubule proteins), vesicle trafficking proteins (Rab proteins and SNARE proteins), and tumor-associated antigens and immune-related proteins [ 92– 99] ( Table 2).

**Table:** **Table 2** Functional characterization of EV surface proteins

EV protein	Key features	Associated diseases	Apply	Ref.
CD9	Communication	Cancers, immune diseases	Immunology, diagnostics	[92]
CD63	Classic marker	Cancers, immune diseases	Detect positioning	[92]
CD81	Communication	Cancers, immune diseases	Functional studies	[92]
HSP70	Involved in the formation of EVs	Cancers	Molecular imaging targets	[93]
HSP90	Involved in the formation of EVs	Cancers	Molecular imaging targets	[94]
Actin	Formation&transportation	Multiple diseases	Molecular imaging targets	[ 95, 96]
Tubulin	Formation& transportation	Multiple diseases	Molecular imaging targets	[ 95, 96]
Rab	Secretion&transport	Multiple diseases	Molecular imaging targets	[ 95, 96]
SNARE	Secretion&fusion	Multiple diseases	Molecular imaging targets	[97]
EGFR	Kinase receptors Immune	Breast cancer	Cancer diagnostics, therapeutic testing, targeted R&D	[98]
PSMA	Specific membrane antigens	Prostate cancer	Cancer diagnosis, early screening	[98]
GPC3	Glycoprotein	Hepatocarcinoma	Cancer diagnosis, early screening	[98]
GPC1	Glycoprotein	Pancreatic cancer	Cancer diagnosis, early screening	[98]
PD-L1	Immune checkpoints	Melanoma, NSCLC, osteosarcoma	Predictive treatment, molecular imaging targets	[99]

Molecular imaging technology has emerged as a powerful noninvasive diagnostic tool capable of detecting various pathologies, including tumors, neurological disorders, and cardiovascular diseases, with high sensitivity. This technique enables *in vivo* visualization of tumor pathophysiological characteristics through targeted molecular probes, proving invaluable for disease diagnosis, treatment monitoring, and evaluation of targeted therapy efficacy. Notably, tumor-derived EV proteins serve as excellent molecular imaging targets. PD-L1 expression in circulating EVs has been established as a monitoring biomarker for melanoma [100], non-small cell lung cancer [101], and osteosarcoma [102] and has the potential to predict immunotherapy response and clinical outcomes [103]. Similarly, EGFR shows promise as an imaging target for breast cancer diagnosis, treatment assessment, and targeted drug development [104]. Furthermore, GPC3, GPC1, and PSMA demonstrate tissue-specific overexpression in hepatocellular carcinoma [105], pancreatic cancer [35], and prostate cancer [106], respectively, making them ideal targets for early cancer detection and therapeutic intervention. EV-associated immune proteins also hold significant diagnostic potential. The ubiquitously expressed CD9 facilitates immunological studies of EVs [107], whereas CD63 serves as a canonical marker for EV detection and localization [108]. CD81 plays crucial roles in EV-mediated intercellular communication and disease pathogenesis [109]. Owing to their essential functions in cellular crosstalk and targeted delivery, these EV proteins are increasingly utilized in molecular imaging studies to enable precise tracking of specific cell populations and tissues.

EV engineering strategies involve two primary approaches: chemical modification and drug loading. Chemical modifications of EVs include click chemistry, noncovalent modifications, and membrane fusion. Smyth *et al*. [110] first demonstrated the application of click chemistry in EV surface functionalization, establishing it as a highly efficient, gentle, and biocompatible modification method. This approach enables rapid and specific conjugation of functional molecules ( *e*. *g*., fluorescent markers, targeting ligands, or drug molecules) to EV surfaces, offering a novel strategy for drug delivery and targeted therapy. Baek *et al*. [111] enhanced the targeting ability of M1-derived EVs against cancer cells through click chemistry-based modifications. Similarly, Kim *et al*. [112] developed a lung cancer-targeted drug delivery system by conjugating aminopolyethylene glycol to EV surfaces via click chemistry. Noncovalent modification, another widely used EV surface engineering technique, offers advantages such as simplicity, high labeling efficiency, strong signal output, and prolonged stability, including ligand-receptor interactions. Han *et al*. [113] functionalized EVs with SIRT6 siRNA, enabling specific recognition by prostate cancer cells and subsequent tumor suppression. Zhang *et al*. [114] employed a guanosine-rich oligonucleotide aptamer (AS1411) to modify EVs, significantly enhancing tumor cell recognition and improving molecular imaging performance. Qi *et al*. [115] developed a breakthrough paramagnetic nanocrystal-based modification of reticulocyte-derived EVs, establishing a tumor-specific ligand-receptor targeting system. This technology significantly enhances delivery precision through selective binding to cellular receptors while minimizing off-target distribution and associated adverse effects. Parallel developments in membrane fusion techniques have expanded EV engineering possibilities. Sato *et al*. [116] pioneered a freeze-thaw plasma membrane fusion method to create EV-liposome hybrids, opening new avenues for nanocarrier design. Subsequent work has demonstrated the therapeutic potential of this approach, with paclitaxel-loaded hybrid EVs showing markedly improved tumor accumulation [117]. These complementary strategies—chemical modification through nanocrystal conjugation and physical modification via membrane fusion—collectively advance the field toward clinically viable EV-based therapeutics.

**Figure 3:**
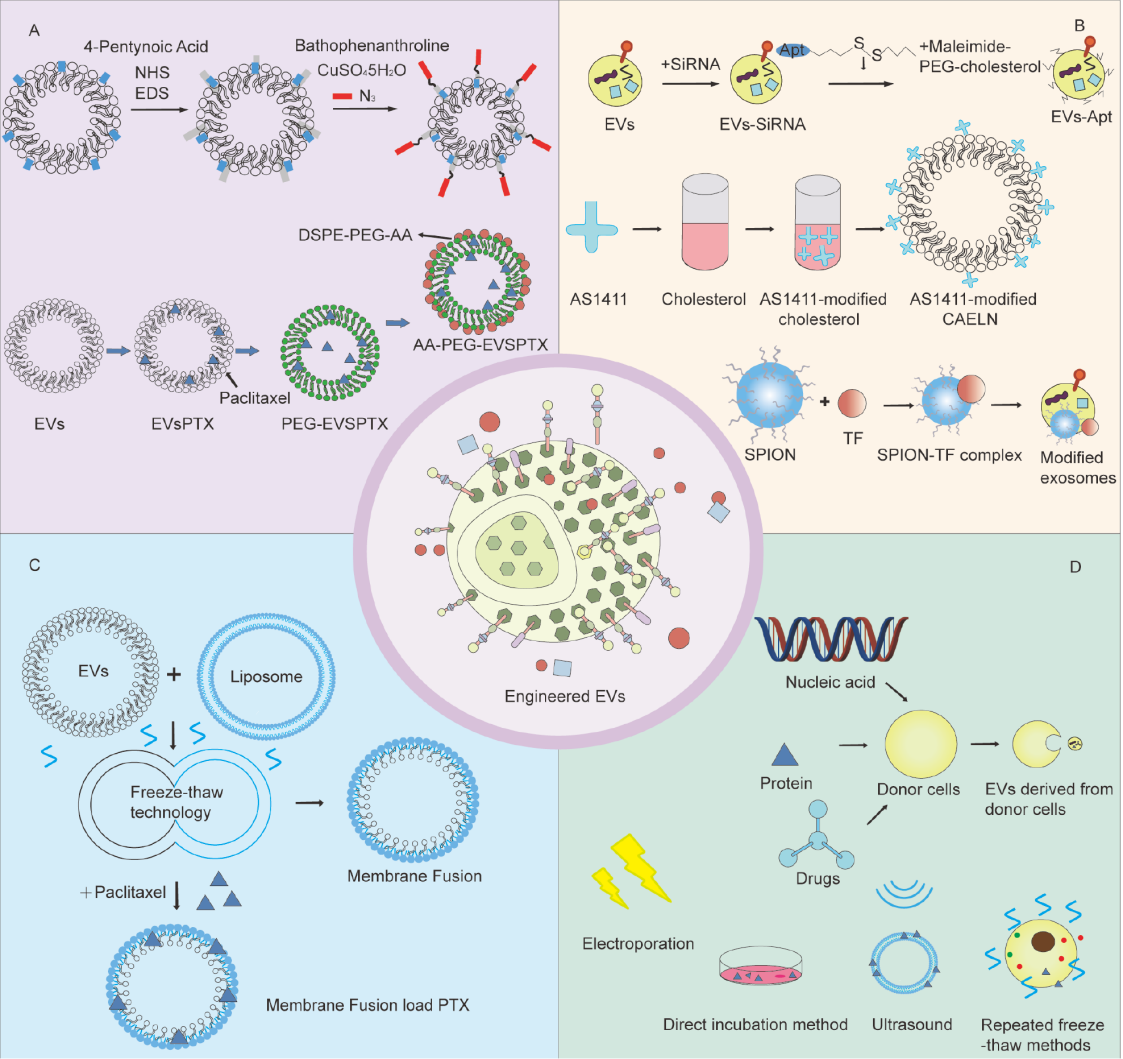
Figure 3 Strategies for loading therapeutic cargo into EVs The illustration depicts multiple engineering approaches, including (A) chemical modification methods using chemical modifiers such as DSPE-PEG-AA and CuSO4/H2O2 to facilitate drug loading and (B) various modification strategies to increase the targeting capability of EVs. These include engineering EVs with SIRT6 siRNA for prostate cancer-specific recognition; functionalizing EVs with AS1411 guanosine-rich oligonucleotide aptamers for improved targeting; employing membrane fusion techniques for direct cargo loading; and conjugating transferrin (TF)-modified superparamagnetic nanocrystals to EV surfaces for magnetically guided delivery. (C) Membrane fusion-mediated paclitaxel loading. (D) Endogenous loading involves introducing target molecules (nucleic acids, proteins, or chemical drugs) into donor cells prior to EV biogenesis, enabling natural packaging during vesicle formation. In addition, there are exogenous drug loading methods, such as electroporation, direct incubation, sonication and cyclic freezing and thawing.

Currently, they can be categorized into endogenous loading and exogenous loading on the basis of the loading strategy. The endogenous loading pathway involves the introduction of target molecules ( *e*. *g*., nucleic acids, proteins, and chemical drugs) into donor cells first by treatment or coincubation with genetic engineering techniques, followed by packaging into EVs. Next, the EVs are secreted from the donor cells, and finally, the drug-loaded EVs are recovered by isolation and purification. Erana-Perez *et al*. [118] successfully achieved imaging and monitoring of EVs via EVs loaded with chimeric proteins such as GFP and luciferase. Abreu *et al*. [119] successfully achieved imaging and monitoring of EVs via bioorthogonal click chemistry to link EVs to specific peptides and molecules, which increased the targeting efficiency and uptake of EVs into target cells. Wiklander *et al*. [41] used molecular engineering tools to bind EVs to the Fc fragment of an antibody, which enabled the modification of different types of immunoglobulin G (IgG), ultimately enabling the targeting of all tumor cells of interest. In addition, endogenously loaded EVs can also be used to deliver chemotherapeutic drugs. Lv *et al*. [120] synthesized genetically engineered EV-thermoliposome hybrid nanoparticles (gETL-NPs) by endogenous loading and showed that these nanoparticles were able to efficiently penetrate tumor tissues and exert therapeutic effects after intravenous injection; however, experiments involving endogenous loading were more difficult, and the cycle time was longer. In addition to endogenous loading, exogenous loading is a common method in which drug molecules are loaded into the interior or on the surface of EVs by physical or chemical means. Common exogenous loading methods include electroporation [121], direct incubation [122], sonication [123], and cyclic freezing and thawing [124], among others. Cotto *et al*. [125] utilized the near-infrared dye indocyanine green (ICG) to load EVs, which showed excellent performance in fluorescence imaging (FLI) and photoacoustic imaging, particularly in the multimodal imaging of oral cancer, which presented good fluorescence intensity and imaging effects. Wang *et al*. [126] loaded natural agonist cyclic dinucleotides (CDNs) onto EVs, which achieved efficient delivery of CDNs in tumor cells and effectively enhanced antitumor immune responses by activating the STING pathway. Compared with endogenous loading, exogenous loading is simpler to perform, such as electroporation, which is highly efficient and utilizes brief high-voltage pulses to create tiny pores in the EV membrane for drug molecules to enter, although it requires certain equipment and operational precision; ultrasonication, which disturbs the structure of the EV membrane to promote the loading of drugs via the cavitation effect generated by ultrasonic waves; and direct coincubation, which is the most convenient method, in which the EVs are incubated with the drug in a suitable environment, and the drug loading process is driven by the concentration gradient. Moreover, a comprehensive analysis of the above imaging methods revealed that EVs have a wide range of potential applications in molecular imaging. By loading fluorescent, bioluminescent or radioactive tracers, real-time monitoring and diagnosis of tumors can be achieved. For example, by labeling EVs with fluorescent dyes or fluorescent proteins for *in vivo* and *ex vivo* imaging; by using luciferase [127] to react with the substrate to generate light signals, information on the dynamic distribution of EVs *in vivo* can be obtained; and by using radiotracer EVs and combining them with SPECT or PET imaging, highly sensitive imaging results can be obtained ( Figure 3).

As research progresses, significant attention has shifted toward evaluating the safety and stability profiles of engineered extracellular vesicles. Two critical aspects demand particular consideration. First, rigorous biocompatibility assessment is essential to ensure that modified EVs maintain immunological properties comparable to those of their native counterparts, thereby preventing excessive immune activation. Second, the pharmacokinetic behavior of drug-loaded EVs must be carefully characterized to guarantee payload stability during systemic circulation, ensuring sustained therapeutic concentrations until targeted tumor delivery is achieved. These considerations underscore the need for comprehensive preclinical evaluation of engineered EV formulations, addressing both immunological compatibility and drug release kinetics to facilitate successful clinical translation.

## Conclusions and Perspectives

EVs have emerged as a promising class of bionanoparticles owing to their inherent biocompatibility and capacity for biomolecule encapsulation. Its therapeutic potential has been particularly recognized in oncology, with demonstrated efficacy against various malignancies, including breast cancer, lung cancer, melanoma, and colorectal carcinoma. Growing evidence further underscores their utility in neurological disorder management and inflammation modulation. However, several critical challenges hinder its clinical translation. While current molecular imaging techniques can track EV biodistribution and clearance kinetics, they suffer from inadequate spatial resolution and sensitivity for precise *in vivo* monitoring at therapeutic doses. Biofunctionalization processes for therapeutic payload or contrast agent loading remain technically demanding, typically yielding less than 30% loading efficiency with poor interbatch reproducibility. Scalable manufacturing faces substantial hurdles, including the lack of standardized good manufacturing practice-compliant protocols, significant batch-to-batch variability from donor cell heterogeneity, and undefined quality control metrics for therapeutic-grade EVs. Additionally, comprehensive characterization of long-term stability under different storage conditions (particularly comparing frozen versus lyophilized formulations) remains incomplete. Although EVs exhibit favorable immunogenicity profiles relative to synthetic nanocarriers, their potential to trigger innate immune responses requires more systematic investigation. While early-phase clinical trials have validated the proof-of-concept for natural EVs, engineered EV products face additional regulatory hurdles concerning product characterization and quality assurance. Overcoming these limitations will necessitate coordinated progress in fundamental vesicle biology alongside innovative bioengineering approaches.
